# Schistosomiasis and Soil Transmitted Helminthiasis Among School Age Children: Impact of 3–5 Annual Rounds of Mass Drug Administration in Ekiti State, Southwest Nigeria

**DOI:** 10.3390/tropicalmed10040085

**Published:** 2025-03-23

**Authors:** Solomon Monday Jacob, Jan-Carel Diehl, Gleb Vdovine, Temitope Agbana, Samuel Popoola, Satyajith Jujjavarapu, David Bell, Akande Oladimeji Ajayi, Joseph O. Fadare, Adebowale F. Akinwumi, Saheed Animashaun, Francisca Olamiju, Moses Oluwaseun Aderogba, Louise Makau-Barasa

**Affiliations:** 1 NTD Division, Federal Ministry of Health, Abuja 900242, Nigeria; 2 Faculty of Industrial Design Engineering, Delft University of Technology, 2628 CE Delft, The Netherlands; j.c.diehl@tudelft.nl (J.-C.D.); gleb.vdovin@gmail.com (G.V.); 3 AiDx Medical BV, 2641 KM Pijnacker, The Netherlands; greatsampop@gmail.com (S.P.); satyajith@aidx-medical.com (S.J.); 4 Independent Consultant, Lake Jackson, TX 77566, USA; bell00david@gmail.com; 5 College of Medicine, Ekiti State University, Ado Ekiti 362103, Nigeria; dejiajayi2@gmail.com (A.O.A.); fadare@eksu.edu.ng (J.O.F.); eyitope.amu@eksu.edu.ng (A.F.A.); 6 Community and Family Health Department, Ekiti State Ministry of Health, Ado Ekiti 360101, Nigeria; saheeday99@gmail.com; 7 Mission to Save the Helpless (MITOSATH), Jos 930001, Nigeria; olamijufo@mitosath.org; 8 The Ending Neglected Diseases (END) Fund, New York, NY 10016, USA; maderogba@endfund.org (M.O.A.); lmakau-barasa@endfund.org (L.M.-B.)

**Keywords:** *Schistosoma haematobium*, *Schistosoma mansoni*, soil-transmitted helminthiasis, mass drug administration, granular, assessment, prevalence, Ekiti, Nigeria

## Abstract

**Background:** Schistosomiasis (SCH) and soil transmitted helminthiasis (STH) have been targeted for elimination as a public health problem (EPHP) within the World Health Organization (WHO)’s Roadmap for Neglected Tropical Diseases (NTDs) 2021–2030. One of the global strategies for the control and elimination of these diseases is the mass administration of praziquantel and albendazole/mebendazole without prior individual diagnosis. To measure the progress towards the 2030 target, we conducted an assessment to determine the impact of the 3–5 rounds of annual mass drug administration among school age children in Ekiti State. Such scientific insights into the impact of these treatments will facilitate improved planning and targeting of resources towards reaching the last mile. **Methodology**: This assessment was conducted in 16 local government areas (LGAs) of Ekiti State between October and November 2023. Samples were collected from pupils in 166 primary and junior secondary schools across 166 wards of the State. Urine and stool samples were collected from 7670 pupils of ages 5 to 14 years, following standard laboratory procedures. Urine membrane filtration techniques were used for urine preparation while the Kato–Katz technique was used for stool preparation. A novel AiDx digital microscope was used to examine the presence of any ova in the prepared specimen. Parasite ova in urine were reported as the number of ova/10 mL of urine, and were categorized as light infection (˂50 ova/10 mL of urine) or heavy infection (>50 ova/10 mL of urine) while ova of parasites in stool samples were reported as eggs per gram of stool (EPG) and categorized into light, moderate and heavy infection. **Results**: Overall, 0.76% (0.56–0.95) at 95% CI of the 7670 respondents were infected with *Schistosomia haematobium*. No *Schistosoma mansoni* infection was recorded in the study. Similarly, 3.9% (3.43–4.29) at 95% CI were infected with STHs. The overall prevalence of schistosomiasis had significantly reduced from 8.2% in 2008 to 0.8%, while the overall prevalence of STHs significantly reduced from 30.9% to 3.9% with *Ascaris lumbricoides* being the dominant species of STH. In the 16 LGAs assessed, Ekiti West had the highest *S. haematobium* prevalence of 4.26%. Ise/Orun and Oye ranked second and third with a prevalence of 3.48% and 2.40% respectively, while all other LGAs had <1% prevalence. The prevalence of STHs was highest in Ekiti-West with a prevalence of 10.45% while Emure and Ikole Local Governments had the lowest prevalence of 0.31% and 0.38%, respectively. There was no significant difference in the prevalence of schistosomiasis between male (0.76%) and female (0.75%) as *p* ≥ 0.05. Similarly, the difference in prevalence for STH among males (3.95%) was not significantly different from their female counterparts (3.77%), *p* ≥ 0.05. **Conclusions:** Based on the WHO guidelines, this study demonstrated that only three LGAs require continued MDA every 2/3 years, seven require only surveillance while six are now non-endemic for schistosomiasis. Similarly, two of the LGAs require one round of MDA yearly, eight LGAs need one round of MDA every two to three years and six LGAs are now below the treatment threshold and no longer require treatment for STH.

## 1. Introduction

Schistosomiasis is a snail-borne acute and chronic parasitic disease that is caused by trematode blood flukes of the genus Schistosoma [1,2]. Globally, it is one of the World Health Organization’s (WHO) neglected tropical diseases (NTDs) that has increasingly drawn the attention of public health experts over the past decade and a half [3]. Transmission has been reported from 78 countries, the majority of which are classified as low- or middle-income countries [3]. An estimated 207 million people in 74 countries are infected with the bulk of the global prevalence (90%) occurring in sub-Saharan Africa [2,3,4]. There are two major forms of schistosomiasis—intestinal (due to *Schistosoma mansoni* and *S. japonicum*) and urogenital (predominantly due to *S. haematobium*) [2]. Both the intestinal form (caused by S. *mansoni*) and the urogenital form (caused by *S. haematobium*) are known to occur in Nigeria, which has the largest number of people in the world in need of treatment (>25 million) [4]. Common signs and symptoms of urogenital S. *haematobium* include a swollen belly, blood in the urine, stunted growth, cognitive impairment in children and infertility among adults of childbearing age. Advanced disease may sometimes be present with fibrosis of the bladder and ureter, kidney damage and bladder cancer [5]. People are often infected during routine agricultural, domestic, occupational (e.g., car washing, sand harvesting, fishing), and recreational activities, which expose them to contaminated waters. Similarly, in tropical and subtropical regions, soil-transmitted helminth (STH) infections are the most widespread neglected tropical diseases (NTDs), especially in places with limited health care, unsafe water sources, and poor sanitation [6,7]. *Ascaris lumbricoides, Trichiuris trichiura*, and hookworm (*Ancylostoma ceylanicum, Ancylostoma duodenale* and *Necator americanus*) species are commonly termed as STHs. This disease of poverty is most common in Sub-Saharan Africa, Asia, and South America [8,9]. Approximately two billion people are currently infected with one or more species of STH, and each year over 4 billion people are at risk of infection, resulting in 4.94 million years of life lost due to disabilities caused by STH. Nigeria has the fourth-largest number of children in need of treatment for STHs (>48 million) [10].

Schistosomiasis and STH have been targeted for elimination as a public health problem (EPHP) within the World Health Organization (WHO)’s Roadmap for Neglected Tropical Diseases (NTDs) 2021–2030 [11]. The cornerstone of current schistosomiasis and STH control is preventive chemotherapy (PC) where praziquantel and albendazole/mebendazole are distributed annually among school age children for mass treatment of schistosomiasis and STH, respectively. The frequency of treatment is determined by the disease endemicity which are classified using parasitological prevalence and intensity of infections [10,12]. The WHO framework recommends that routine monitoring for effective coverage and evaluation of the impact of intervention using repeat population-based surveys be conducted after five rounds of preventive chemotherapy, or more frequently with a mid-term evaluation after three rounds as integral parts of preventive chemotherapy programs, to help inform the decision on changing the strategy and continuing or stopping the program.

## 2. Problem Statement

Schistosomiasis (SCH) and soil transmitted helminthiasis (STH) have been targeted for elimination as a public health problem (EPHP) within the World Health Organization (WHO)’s Roadmap for Neglected Tropical Diseases (NTDs) 2021–2030. One of the global strategies for the control and elimination of these diseases is mass administration of praziquantel and albendazole/mebendazole without prior individual diagnosis. To align with this goal, a baseline prevalence and endemicity study for the disease was conducted in 2008 with prevalence ranging from 0.00 to 32% and 20.00 to 48.94% for schistosomiasis and STH, respectively, across various LGAs (the administrative units for treatment decisions), (Table S1). However, treatment did not commence until 2015 when LGAs were stratified to receive mass drug administration (MDA) for schistosomiasis and STH either annually or every other year. The 15 endemic LGAs received albendazole/mebendazole for STH annually from 2015 to 2022 except in 2021 where only Ado Ekiti, Ilejemeje and Irepodun/Ifelodun received treatment. Similarly for schistosomiasis, Ado Ekiti, Ekiti West and Ise/Orun, Ekiti South West and Gbonyi LGAs were targeted for annual treatment with praziquantel while others were targeted for every other year except Irepodun/Ifelodun where it was not endemic. All targeted LGAs received treatment in 2015, 2017, 2019 and 2022. More detailed treatment histories are available in Supplemental Table S2. Administratively, reported treatment coverage among school-age children was skeletal and sub-optimal in 2015 but improved generally to above 75% from 2016. Although coverage dropped in one round because of the shortage of praziquantel and mebendazole tablets, all LGAs achieved between three and five rounds of effective treatment. To measure the progress towards the 2030 target, we conducted an assessment to determine the impact of the 3–5 rounds of annual mass drug administration among school age children, compare the prevalence of the diseases in 2023 to current prevalence, and provide suggestions to improve overall outcomes in Ekiti States, Southwest Nigeria.

### Ethical Clearance

The ethical approval was received from the Research Ethics Committee (Ekiti State Ministry of Health and Human Services, HREC) under approval number: MOH/EKHREC/EA/P/59 and all research was performed in accordance with the relevant guidelines and regulations. Children with severe disease requiring urgent medical intervention, or residing in the area for less than 6 months, were excluded.

## 3. Methodology

### 3.1. Study Site and Population

**Figure 1:**
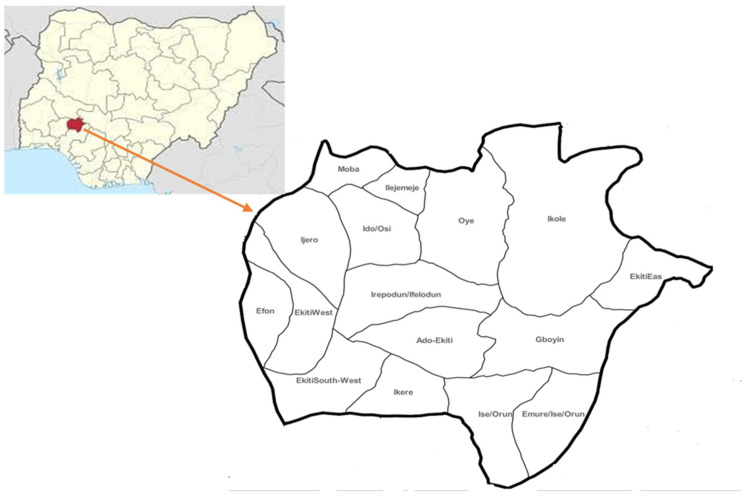
Map of Nigeria showing the study area.

Ekiti State is in the southwestern region of the country. It is located between longitudes 40°51′ and 50°451′ east of the Greenwich meridian and latitudes 70°151′ and 80°51′ north of the Equator (Figure 1). It has a population of 3,480,006 (male−1,774,803 and female−1,705,203), as at 2023 when projected from the 2006 population census, and covers a land area of 5434 square kilometers. It has 16 local government areas, distributed across 3 senatorial zones with a population growth rate of 3.1% per annum.

### 3.2. Study Design: Site Selection and Sample Size

This assessment was conducted in one selected site each from 166 wards of Ekiti State and results aggregated at LGA level to measure any changes in disease burden. It was conducted between 15 October and 20 November 2023, at least 6 months after the last annual round of SCH+STH MDA.

During the 2008 SCH+STH baseline survey, 5 communities were randomly selected from each of the 16 LGAs and prevalence was aggregated as mean of the 5 sites. However, during this impact survey in 2023, the number of communities increased to about 10 with 1 community selected from each political ward. Prevalence was equally aggregated as mean of the 10 sites. This was necessary to obtain data at a more granular level due to heterogeneity in the distribution of schistosomiasis. The study was school-based, targeting school-age children. A two-stage approach was used for selection; in the first stage, schools were purposively selected guided by previous knowledge of sites where the baseline survey in 2008 were conducted. The added communities were selected based on where transmission is known, suspected or more likely near water bodies. In the second stage, 50–55 participants, age ranging from 5 to 14 years were selected using a systematic random sampling frame as described by [13,14].

### 3.3. Stool and Urine Sample Collection at School

On the morning of sample collection, after obtaining consent and assent, participants were provided with a labeled wide mouthed stool container, a piece of plain paper, a piece of applicator stick and a piece of toilet paper. The participants were instructed to defecate on the piece of paper provided, to avoid contamination from the toilet environment, and then transfer a portion of the stool to the clean plastic container using the applicator stick. Each participant was also issued an empty urine container and other sanitary necessities and instructed to pass the last stream of urine into the bottle. Urine samples were collected between 10.00 am and 2.00 pm. Both samples were returned to the survey team who assigned a unique study identification number, used to track all collected samples. Collected fresh stool and urine samples were then transported within 2 h of collection in cool boxes containing ice packs to the Ekiti State Teaching Hospital Laboratory, where they were processed by urine filtration and Kato–Katz techniques for urine and stool, respectively.

### 3.4. Urine Filtration Technique

In the laboratory, each urine sample was homogenized by gentle agitation and 10 mL was filtered using urine filtration technique to concentrate eggs of schistosome on membrane filter 13 mm and 12 µm pores (Starlitech corporation, Auburn-USA) as described by [15]. Prevalence was reported as the proportion of persons who had infection with the parasite while intensity of *S, haematobium* was reported as the number of ova/10 mL of urine, and were categorized as light infection (˂50 ova/10 mL of urine) or heavy infection (>50 ova/10 mL of urine).

### 3.5. Kato–Katz Technique for Stool Examination

Stool samples were also examined for parasite eggs using the Kato–Katz technique described by [16]. Prevalence was reported as the proportion of persons who had any of the parasites (prev = No. of cases/respondents × 100) while intensity was reported as the number of eggs per grams (epg) of stool and were categorized as light, moderate or heavy infection. An infection with A. lumbricoides was classified as light if there were 1–4999 EPG, moderate if there were 5000–49,999 EPG, and heavy if there were more than 50,000 EPG. Similarly, for *T. trichiura*, a light infection corresponded to 1–999 EPG, moderate to 1000–9999 EPG, and heavy to more than 10,000 EPG. For hookworms, the classifications were as follows: light intensity was defined as 1–1999 EPG, moderate intensity was defined as 2000–3999 EPG, and heavy intensity was defined as more than 4000 EPG.

AiDx NTDx device, a digital microscope with performance accuracy of 95% [17,18] compared to a conventional microscope was used to examine prepared slides. Quality assurance was performed by systematic random examination of 10% of the daily examined slides by a qualified independent laboratory scientist using a conventional microscope.

### 3.6. Data Processing and Analysis

Data were entered into Microsoft Excel and imported into IBM SPSS version 20 which was used for statistical analysis. Descriptive statistics were used to describe the occurrence of infection and the Pearson Chi-square test association between schistosomiasis infection status and other variables like sex. Pearson Chi-square was also used to compare the proportion of infections between communities and LGAs.

## 4. Results

### 4.1. Overall Prevalence of Schistosomiasis and STH

A total of 7670 pupils responded by providing their urine and stool samples. Fifty-eight (0.76% at 95% CI 0.56–0.95) were infected with S. *haematobium* while 296 (3.9% at 95% CI 3.43–4.29) were infected with at least one of the soil-transmitted helminths. No *S. mansoni* infection was recorded in this study. Ekiti West had the highest S. *haematobium* prevalence of 4.26%. Ise/Orun and Oye ranked second and third with a prevalence of 3.48% and 2.40%, respectively. No schistosome was detected in any respondent in Efon, Ekiti-East, Ekiti South-west, Ido/Osi or Moba. Similarly, Ekiti west LGA had the highest prevalence (10.4%) of STH, followed by Gbonyin (9.6%) and the least (0.3%) was recorded in Emure LGA (Table 1).

### 4.2. Prevalence of Schistosomiasis and STH by Sex

The overall distribution of schistosomiasis infection among male respondents (0.76%) was not significantly different from the female respondents (0.75%) (*p* > 0.05). However, specific LGAs such as Ekiti West showed a significantly higher prevalence (*p* < 0.05) among females (5.62%) than males (2.73%). In contrast, Ise/Orun LGA showed a significantly higher prevalence (*p* < 0.05) among males (4.47%) than among females (2.21%) (Table 2). Similarly, the overall distribution of STH among male respondents (3.95%) was not significantly different from female respondents (3.77%). This trend in the distribution is similar across most of the LGAs except in Oye LGA where the distribution is significantly higher (*p* < 0.05) among males (5.24%) than among females (0.96%) (Table 2).

### 4.3. Distribution of Parasites by Species Across the LGA

**Figure 2:**
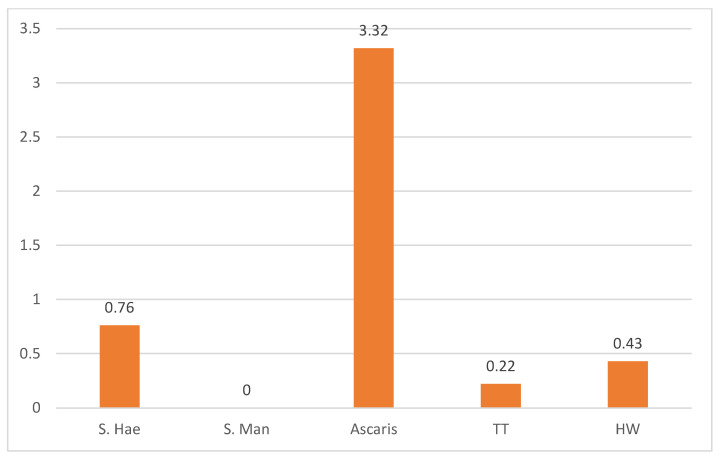
Prevalence of parasites by species.

In terms of parasite distribution by species, out of the 7670 pupils examined, 58 (0.76%) were infected with urinary Schistosoma (S. *haematobium)*, and no intestinal Schistosoma (*S. mansoni*) was recorded. For any form of STHs species, *A Lumbricoides (AL)* constituted the highest proportion with 255 (3.2%) while *Tricuris trichuria (TT)* and hookworm (HW) recorded lower prevalence of 0.22% and 0.42% respectively, (Figure 2).

### 4.4. Infection Load Estimation

Intensity of infection for schistosomiasis was predominantly light, at 89.7%; the few (0.8%) who were infected with the parasite had light infection. Heavy intensity of infection was only observed among six (10.3%) of those infected. Similarly, 98.6% of those infected with STH had light infection while only 4 (1.4%) of the 296 who were positive for any form of the STH had moderate infection and none with heavy infection based on WHO classification of intensity. The difference in the level of intensity for both schistosomiasis and STH across the different LGA were not significantly different as *p* > 0.05 (Table 4).

### 4.5. Prevalence After 2–3 Rounds of Treatment Compared with Baseline Prevalence

**Figure 3:**
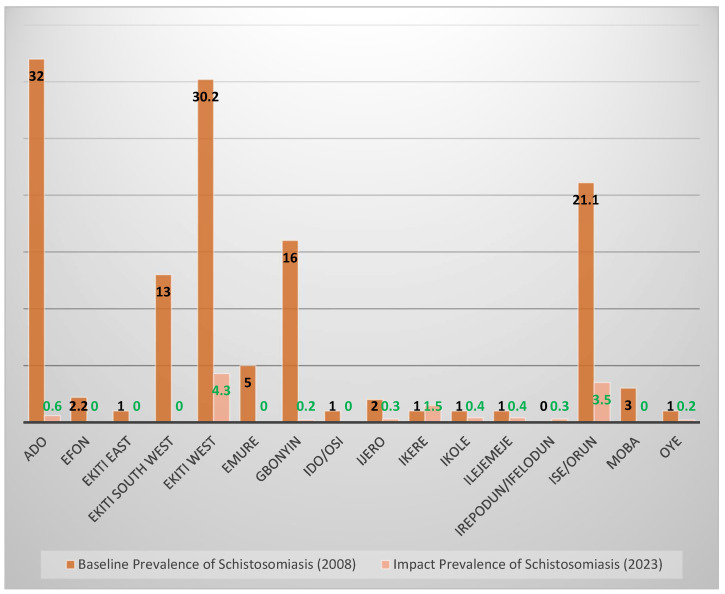
Impact prevalence compared to baseline prevalence for schistosomiasis where deeper brown = baseline prevalence and light brown = impact prevalence.

Figure 3 below compares the prevalence of SCH in the 2008 baseline surveys with the prevalence in this impact survey in 2023. Ado Ekiti and Ekiti West demonstrates a clear decline from 32% and 30.2% prevalence level to 0.6% and 4.6%, respectively. Six LGAs, including: Efon, Ekiti East, Ekiti South West, Emure, Ido/Osi and Moba also recorded an impressive decline to 0%.

A marginal increase in the disease from 0% in 2008 to 0.3% in this 2023 impact survey was observed in Irepodun/Ifelodun LGA. All the communities in this LGA demonstrated 0% prevalence except Igbemo with a prevalence of 2.9% leading to a cumulative prevalence of 0.3%. Similar increase in prevalence was observed in Ikere LGA where prevalence has increased from 0% to 0.5%. Again, one community (Ugele) with a prevalence of 13.1% was responsible for this increase.

**Figure 4:**
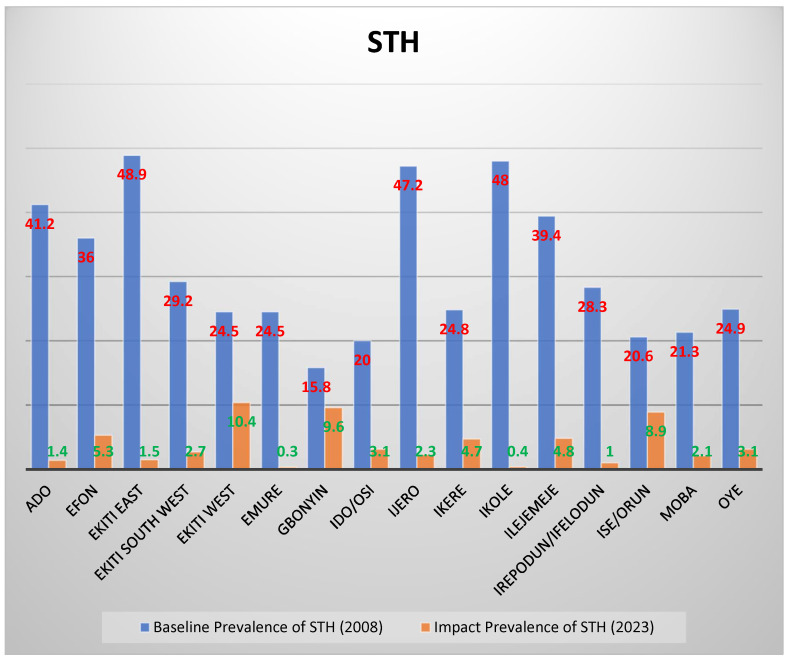
Impact prevalence compared to baseline prevalence for STH where blue colour = baseline prevalence and brown colour = impact prevalence.

Figure 4, below, shows the comparison between the baseline prevalence in 2008 and the 2023 impact study for STH. All the LGAs recorded a significant reduction in prevalence compared to the 2008 baseline figures. Ekiti East and Ikole LGAs with prevalence of 48.9% and 48% at baseline were reduced drastically to 1.5% and 0.4%, respectively.

## 5. Discussion

Soil transmitted helminthiasis and schistosomiasis have been targeted for elimination by 2030 according to the WHO Roadmap [19]. Consequently, each state in Nigeria is working to meet this target by assessing its progress and reviewing its strategies and approach. Significant gaps in epidemiological data create difficulties in understanding the true distribution of the disease and necessary adjustments [20]. Therefore, a recommended approach to understanding the progress made in the control of these diseases is to conduct an impact assessment after rounds of effective treatment [21].

We observed a significant decline in the prevalence and intensity of schistosomiasis and STH among school-aged children after 3–5 years of mass drug administration across LGAs in Ekiti State. Although the baseline study was conducted in 2008, no intervention took place until 2015 when mass treatment was instituted as the only control measure. The study followed the same methodology as that of 2008 using urine membrane filtration and Kato–Katz techniques, targeting 50–55 school age children from each community. However, while an average of five sites were selected from each LGA in the 2008 study, this impact study added more sites to ensure that treatment impact was not limited to the baseline communities only. The results from each study were aggregated at LGA level and provided the overall endemicity at each LGA level. The overall prevalence of schistosomiasis has significantly reduced from 8.2% in 2008 to 0.8% in this study, following 3–5 rounds of annual treatment with praziquantel. This finding indicates effectiveness of the deworming program in the states despite imperfect implementation. Other factors such as water, sanitation and hygiene practices (WASH) are known to significantly impact the burden of these diseases [22,23,24]. However, preventive chemotherapy was the only control strategy in the study area as the WASH conditions predominantly remain the same as in 2008. Ado-Ekiti (state capital), with a baseline prevalence of 32% in 2008, is now reduced to 0.6%. This reduction in endemicity supports the findings of [25,26] that praziquantel administered at a single oral dose of 40 mg/kg should achieve a cure rate of 89.8–91.7% and in individuals not cured, the drug should cause egg excretion to be reduced by 86.8–91%. Interestingly, *Schistosoma haematobium,* the urogenital form accounts for all the schistosomiasis prevalence observed in this study as no single *S. mansoni,* the intestinal form, was seen. This is consistent with a previous study that *s. mansoni* is less prevalent in Ekiti State [27].

Although the overall prevalence has been reduced to less than 1%, Ekiti West, Ise/Orun and Ikere, still had prevalence as high as 4.3%, 3.5% and 1.5%, respectively. Going by the WHO threshold of 1% to stop treatment, all the LGAs in Ekiti State apart from these three LGAs no longer require any form of mass treatment for schistosomiasis but surveillance. The three LGAs with prevalence greater than 1% still require one round of mass treatment every 2–3 years.

A marginal increase in the prevalence of schistosomiasis was observed in some LGAs that were classified as not endemic from the baseline survey of 2008 and had not received any form of mass treatment. The movement of people or animals across LGAs and from one local community may have introduced the disease into these communities. However, since the endemicity is still below the 1% treatment threshold, no mass administration of praziquantel is required for these LGAs but identified hotspot communities with prevalence greater than 1% will require mass treatment.

Similarly, the overall prevalence of STHs has significantly reduced from 30.9% in the 2008 baseline survey to 3.9% in this study with *Ascaris lumbricoides* as the dominant species of STH in the state. This is consistent with previous research suggesting that *A. lumbricoides* contributes significantly to the global burden of parasitic infections due to its robust egg production and survival capacity in varying environmental conditions [28,29]. This finding showed that six LGAs now have prevalence below the 2% treatment threshold which qualifies them to stop treatment and move to surveillance phase where only targeted treatment is required. Prevalence for the other 10 LGAs now lies between 2 and 10% and will require continuation of one round of MDA annually.

There was no significant difference in the distribution of infection among males and females with *p* > 0.05 for both schistosomiasis and STH. This indicates that both genders are equally exposed to the same risk factors which may include availability and non-availability of water bodies and toilet facilities within the school environment.

The prevalence of light and heavy-intensity infections for S. haematobium between the LGAs was not significantly different across the State (*p* > 0.05). This shows that while children in some communities may have higher mean egg counts indicating more intense infection, the distribution of infection intensity levels, whether light or heavy, remains similar across all LGAs. This could imply the homogeneity nature of the LGAs which are predominantly rural and experience comparable exposure risk, particularly in areas lacking adequate public health measures and sanitation practices to prevent disease transmission. The predominantly low intensity coupled with the low prevalence indicates that these diseases have been reduced to a level where they are no longer of public health risk in the state. However, surveillance will be required to completely eliminate the disease from the state.

The use of a novel digital microscopy which was easy to use in both the laboratory and the field setting reduced the sample analysis time with less human manipulation. Such devices could be deployed to rural communities that lack adequate manpower and expertise to improve the efficiency and make such surveys feasible [17,18].

It took more than a decade of intervention with MDA to achieve this modest progress. It underscores the fact that MDA alone cannot bring about the elimination of schistosomiasis and STH without the successful implementation of water, sanitation, and hygiene (WASH) practices and behavioral change intervention [22,23,24]. To sustain these gains, it is important to sustain the provision of potable water in those communities to reduce continuous exposure to sources of infection and break the transmission cycle. Community engagement and the implementation of behavior change campaigns will enhance the sustainability of the gains recorded and progress toward the 2030 elimination targets.

## 6. Limitations of the Study

This study was unable to collect specimens from some communities due to misconception and religious beliefs and where samples were already collected, the survey teams were forced to return the sample. The results provided in this study do not include such communities.

## 7. Conclusions

The prevalence and intensity of schistosomiasis and STH in Ekiti State have been significantly reduced from what they were in 2008 to a level where they are no longer a public health problem in most of the LGAs, underscoring the impact of the 3–5 rounds of annual mass treatment. However, further effort is required, including investments in vector control, clean water and sanitation (WASH) interventions, to achieve elimination. Both human and material resources can be concentrated more effectively in the few areas that still require treatment to meet the 2030 elimination target.

## Figures and Tables

**Table 1:** Overall prevalence of Schistosomiasis and STH.

LGA	No. of Respondents	Prevalence of Schistosomiasis			Prevalence of STH		
		No. Positive	Prev. %	95% CI	No. Positive	Prev. %	95% CI
Ado	658	4	0.6	0.14–1.20	9	1.4	0.48–2.26
Efon	416	0	0	0.00	22	5.3	3.13–7.44
Ekiti East	618	0	0	0.00	9	1.5	0.51–2.40
Ekiti South West	556	0	0	0.00	15	2.7	1.35–4.04
Ekiti West	469	20	4.3	2.44–6.09	49	10.4	7.68–13.22
Emure	320	0	0	0.00	1	0.3	‒0.30–0.90
Gbonyin	447	1	0.2	0.02–0.60	43	9.6	6.89–12.35
Ido/Osi	509	0	0	0.00	16	3.1	1.63–4.66
Ijero	303	1	0.3	0.03–0.98	7	2.3	0.62–4.00
Ikere	550	8	1.5	0.40–2.46	26	4.7	2.95–6.50
Ikole	527	2	0.4	0.10–0.90	2	0.4	0.15–0.90
Ilejemeje	546	2	0.4	0.14–0.87	26	4.8	2.98–6.55
Irepodun/Ifelodun	396	1	0.3	0.02–0.70	4	1	0.03–2.00
Ise/Orun	517	18	3.5	1.90–5.10	46	8.9	6.44–11.35
Moba	420	0	0	0.00	8	1.9	0.60–3.21
Oye	418	1	0.2	0.23–0.71	13	3.1	1.45–4.77
Total	7670	58	0.8	0.56–0.95	296	3.9	3.43–4.29

**Table 2:** Overall prevalence of schistosomiasis and STH by sex.

Disease	Total Sampled	Male		Female		*p*-Value
		Sampled	No. Positive (Prevalence)	Sampled	No. Positive (Prevalence)	
Schistosomiasis	7670	3823	29 (0.76%)	3847	29 (0.75%)	*p* > 0.05
STH	7670	3823	151(3.95%)	3847	145 (3.77%)	*p* > 0.05

**Table 4:** Intensity of parasite infection.

LGA	No. Examined	Schistosomiasis			STH			
		No. Positive	Light Infection (< 50 Eggs per mL)	Heavy Infection (> 50 Eggs per mL)	No. Positive	Light Infection (1–999 epg)	Moderate Infection (1000–9999 epg)	Heavy Infection (≥10,000 epg)
Ado	658	4	3 (75%)	1 (25%)	9	9 (100%)	0 (0%)	0 (0%)
Efon	416	0	0 (0%)	0 (0%)	22	22 (100%)	0 (0%)	0 (0%)
Ekiti East	618	0	0 (0%)	0 (0%)	9	9 (100%)	0 (0%)	0 (0%)
Ekiti South West	556	0	0 (0%)	0 (0%)	15	15 (100%)	0 (0%)	0 (0%)
Ekiti West	469	20	19 (95%)	1 (5%)	49	47 (96%)	2 (4%)	0 (0%)
Emure	320	0	0 (0%)	0 (0%)	1	1 (100%)	0 (0%)	0 (0%)
Gbonyin	447	1	1 (100%)	0 (0%)	43	43 (100%)	0 (0%)	0 (0%)
Ido/Osi	509	0	0 (0%)	0 (0%)	16	16 (100%)	0 (0%)	0 (0%)
Ijero	303	1	1 (100%)	0 (0%)	7	7 (100%)	0 (0%)	0 (0%)
Ikere	550	8	7 (87.5%)	1 (12.5%)	26	25 (96%)	1 (4%)	0 (0%)
Ikole	527	2	2 (100%)	0 (0%)	2	2 (100%)	0 (0%)	0 (0%)
Ilejemeje	546	2	2 (100%)	0 (0%)	26	26 (100%)	0 (0%)	0 (0%)
Irepodun/Ifelodun	396	1	0 (0%)	1 (100%)	4	3 (75%)	1 (25%)	0 (0%)
Ise/Orun	517	18	16 (89%)	2 (11%)	46	46 (100%)	0 (0%)	0 (0%)
Moba	420	0	0 (0%)	0 (0%)	8	8 (100%)	0 (0%)	0 (0%)
Oye	418	1	1 (100%)	0 (0%)	13	13 (100%)	0 (0%)	0 (0%)
TOTAL	7670	58	52 (89.7%)	6 (10.3%)	296	292 (98.6%)	4 (1.4%)	0 (0.0%)

*p* > 0.05.

## Data Availability

The original contributions presented in this study are included in the article/supplementary material. Further inquiries can be directed to the corresponding authors.

## Supplementary Materials

The following supporting information can be downloaded at: https://www.mdpi.com/article/10.3390/tropicalmed10040085/s1, Table S1: Baseline prevalence of schistosomiasis and STH; Table S2: Treatment coverage from 2015-2022; Table S3: Prevalence of Schistosomiasis by Sex; Figure S1: Decision tree on Schistosomiasis control; Figure S2: Decision tree on STH control.
