# Supplementary material for: Schistosomiasis and Soil Transmitted Helminthiasis Among School Age Children: Impact of 3–5 Annual Rounds of Mass Drug Administration in Ekiti State, Southwest Nigeria
Source: Trop Med Infect Dis. 2025 Mar 23;10(4):85. doi: 10.3390/tropicalmed10040085 (PMC12030984; doi:10.3390/tropicalmed10040085)
Supplement: Supplementary file 1 [file tropicalmed-10-00085-s001.zip › tropicalmed-3472577-supplementary.pdf]

Supplementary Table S1: Baseline prevalence of schistosomiasis and STH

	SCHISTOSOMIASIS		STH	
LGA	LEVEL OF ENDEMICITY (%)	RECOMMENDED TREATMENT PLAN	LEVEL OF ENDEMICITY (%)	RECOMMENDED TREATMENT PLAN
**Ado Ekiti**	**32**	Once yearly	**41.2**	Once yearly
**Efon**	**2.2**	Once in 2 yrs	**36.0**	Once yearly
**Ekiti East**	**1**	Once in 2 yrs	**48.9**	Once yearly
**Ekiti South West**	**13**	Once yearly	**29.2**	Once yearly
**Ekiti West**	**30.2**	Once yearly	**24.5**	Once yearly
**Emure**	**5**	Once in 2 yrs	**24.5**	Once yearly
**Gbonyin (Aiyekiri)**	**16**	Once yearly	**15.8**	No treatment
**Ido-Osi**	**1**	Once in 2 yrs	**20.0**	Once yearly
**Ijero**	**2**	Once in 2 yrs	**47.2**	Once yearly
**Ikere**	**1**	Once in 2 yrs	**24.8**	Once yearly
**Ikole**	**1**	Once in 2 yrs	**48.0**	Once yearly
**Ilejemeje**	**1**	Once in 2 yrs	**39.4**	Once yearly
**Irepodun/Ifelodun**	**0.0**	No treatment	**28.3**	Once yearly
**Ise/Orun**	**21.1**	Once yearly	**20.6**	Once yearly
**Moba**	**3**	Once in 2 yrs	**21.3**	Once yearly
**Oye**	**1**	Once in 2 yrs	**24.9**	Once yearly

Supplementary Table S2: Treatment coverage from 2015-2022

LGA	MDA THERAPEUTIC COVERAGES FOR SCHISTOSOMIASIS									MDA THERAPEUTIC COVERAGES FOR STH (%)							
	2010	2015	2016	2017	2018	2019	2020	2021	2022	2015	2016	2017	2018	2019	2020	2021	2022
Ado Ekiti		25	53		20	83	93		97	4.3	55.3	63.9	71	68.0678	100	104	118
Efon		15	30	100	-	92			90	23.2	68.5	97.5	82	75.31589	93		89
Ekiti East	70	100		100	27	71			94	64.9	100.0	100.0	100	94.78646	100		116
Ekiti South West	68	79			20	51	66		88	25.3	79.8	93.5	100	87.74298	85		84
Ekiti West	66	20	34		19	100	100		86	9.9	74.4	95.4	85	84.27354	77		104
Emure		15	38		-	36			89	32.8	77.2	84.4	100	0	70		112
Gbonyin (Aiyekiri)	173	100			29	100	94		100	19.0	45.0		-	91.44725			
Ido-Osi		76			25	100			90	4.5	43.7	78.3	83	90.9156	95		103
Ijero		65		94	-	100			88	44.2	70.8	95.4	100	100	82		101
Ikere	4	74			14	100			94	19.1	43.7	87.3	81	63.51584	87		87
Ikole	39	89		4	-	100			84	25.4	85.9	86.9	97	86.34231	96		94
Ilejemeje	260	93		100	-	100			87	76.6	74.4	100.0	85	84.53663	100	94	97
Irepodun/Ifelodun	171				-	0				21.7	90.9	100.0	100	97.53044	64	112	108
Ise/Orun		36	35		31	52	100		89	22.6	100.0	100.0	100	83.20237	82		86
Moba	69	79		100	-	100			86	52.3	96.6	100.0	100	97.89541	77		96
Oye		88			29	100			94	36.6	100.0	100.0	100	70.8828	100		95

Supplementary Table S3: Prevalence of Schistosomiasis by Sex

S/N	LGA	No. of Participants (Positive)	No. of Male (Positive)	No. of Female (Positive)	Prevalence of Schistosomiasis among Male	Prevalence of Schistosomiasis Among Female	Prevalence of Schistosomiasis among respondents
1.	Ado	658 (4)	324 (2)	334 (2)	0.62	0.60	0.61
2.	Efon	416 (0)	199 (0)	217 (0)	0.00	0.00	0.00
3.	Ekiti East	618 (0)	313 (0)	305 (0)	0.00	0.00	0.00
4.	Ekiti South West	556 (0)	250 (0)	306 (0)	0.00	0.00	0.00
5.	Ekiti West	469 (20)	220 (6)	249 (14)	2.73	5.62	4.26
6.	Emure	320 (0)	161 (0)	159 (0)	0.00	0.00	0.00
7.	Gbonyin	447 (1)	222 (0)	225 (1)	0.00	0.44	0.22
8.	Ido/Osi	509 (0)	260 (0)	249 (0)	0.00	0.00	0.00
9.	Ijero	303 (1)	169 (1)	134 (0)	0.59	0.00	0.33
10.	Ikere	550 (8)	265 (4)	285 (4)	1.51	1.40	1.45
11.	Ikole	527 (2)	261 (1)	266 (1)	0.38	0.38	0.38
12.	Ilejemeje	546 (2)	258 (0)	288 (2)	0.00	0.70	0.37
13.	Irepodun/Ifelodun	396 (1)	207 (1)	189 (0)	0.48	0.00	0.25
14	Ise/Orun	517 (18)	291 (13)	226 (5)	4.47	2.21	3.48
15.	Moba	420 (0)	213 (0)	207 (0)	0.00	0.00	0.00
16.	Oye	418 (1)	210 (1)	208 (0)	0.48	0.00	2.40
**TOTAL**		**7670 (58)**	**3823 (29)**	**3847 (29)**	**0.76**	**0.75**	**0.76**

Supplementary figure S1: Decision tree on Schistosomiasis control

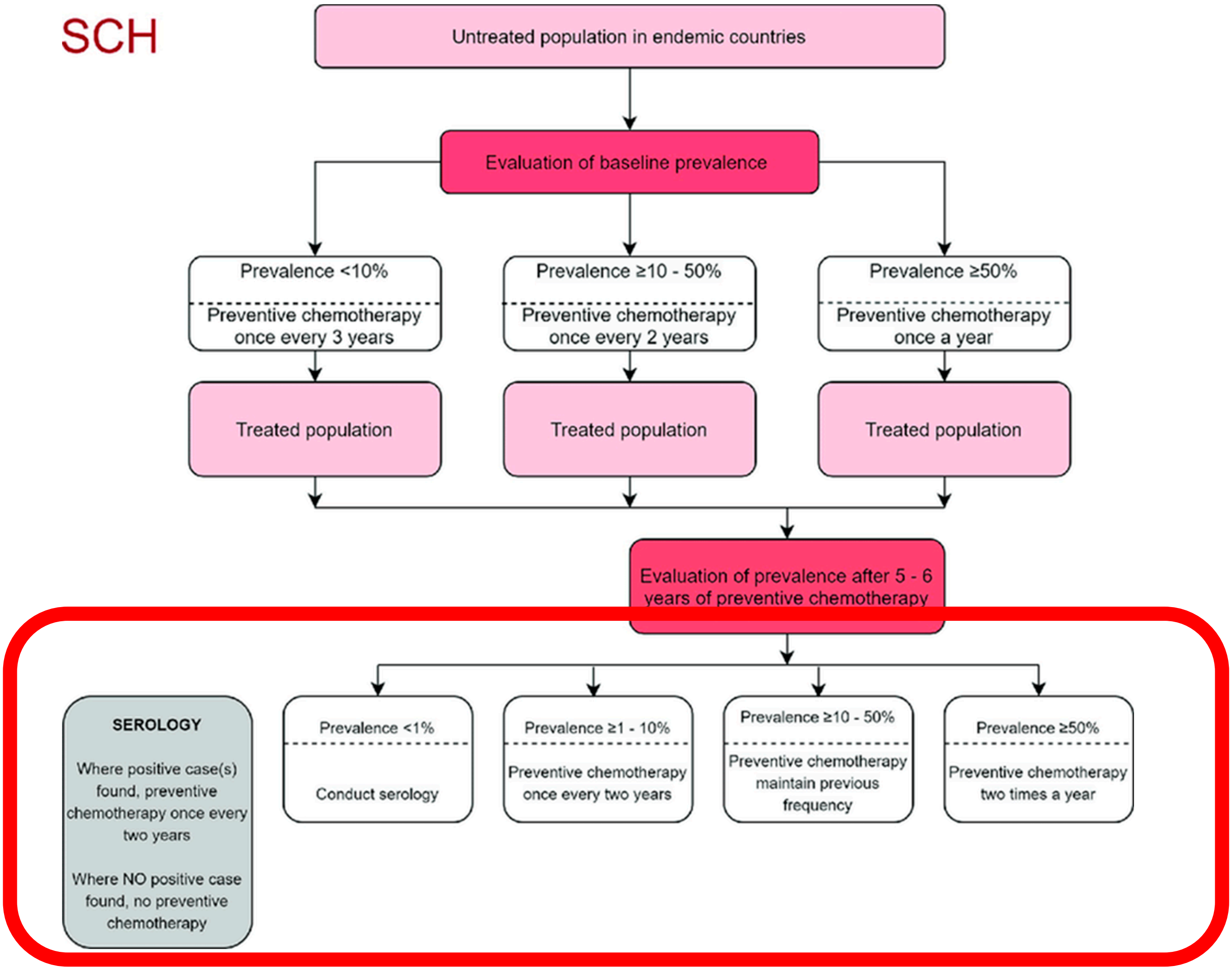

WHO 2011; Helminth control in school-age children

Supplementary figure S2: Decision tree on STH control

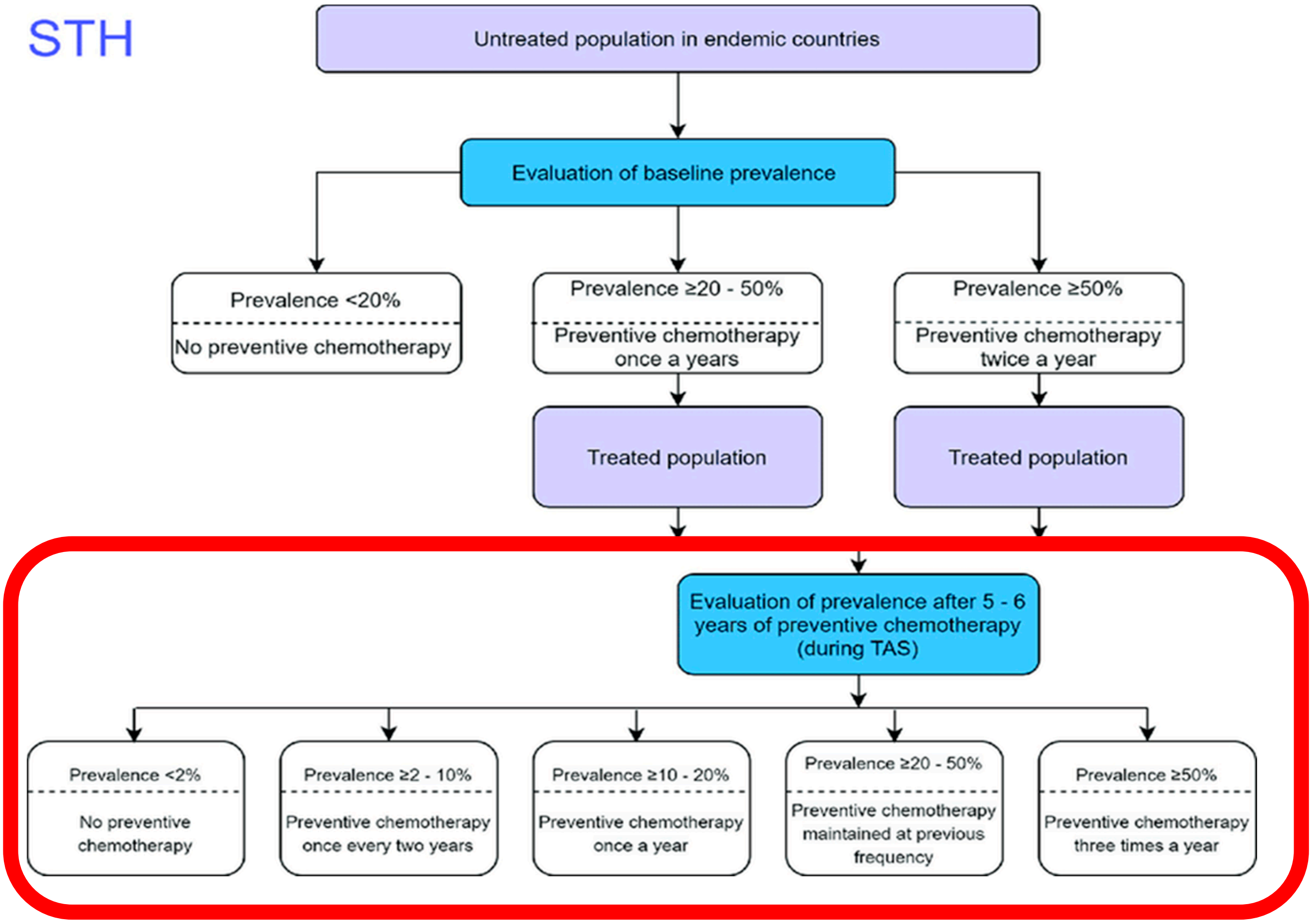

WHO 2011; Helminth control in school-age children.
